# Validity of an immersive virtual reality training system for orthognathic surgical education

**DOI:** 10.3389/fped.2023.1133456

**Published:** 2023-03-23

**Authors:** Teng Wan, Kai Liu, Biao Li, Xudong Wang

**Affiliations:** ^1^ Department of Oral and Craniomaxillofacial Surgery, Ninth People’s Hospital, Shanghai Jiao Tong University School of Medicine, Shanghai, China; ^2^ Shanghai Key Laboratory of Stomatology & Shanghai Research Institute of Stomatology, National Clinical Research Center of Stomatology, Shanghai, China

**Keywords:** immersive virtual reality, orthognathic surgery, surgery training, validity, surgical education and training

## Abstract

Virtual reality (VR) has been proven an important supplement for surgical education in medical students. However, studies on immersive VR (iVR) simulation in orthognathic surgical education are limited. This study aimed to assess the validity of the iVR surgical training system for orthognathic surgery. Participants completed questionnaires at the end of the course to assess the validity of the training system. The questionnaires included questions on the experience of using the iVR system and surgical authenticity. Seven experienced surgeons and seven inexperienced students were recruited in this study to use our self-developed iVR training system for orthognathic surgery. The participants showed strong agreement to the fidelity of our training system (4.35 out of 5), including the virtual environment, instruments, anatomy structures, and surgical procedures. The participants also strongly agreed that the iVR technique was essential in imparting surgical education. However, most of the participants experienced some degree of dizziness or fatigue after 1 h of using the system. The iVR training system is a new method for imparting education about orthognathic surgery. The iVR training system can act as a supplement and potential substitute of the traditional surgical training method.

## Introduction

Surgical residents commonly acquire new procedure knowledge through a combination of reading technique guides and watching surgical procedure videos (1, 2).

In recent years, significant advancements in computer sciences have enabled the development of innovative training tools in the medical field, which has enhanced the knowledge and technical skills acquired by surgical trainees (3).

Virtual reality (VR) simulator training has enabled trainees to learn surgical skills in a risk-free environment while also increasing their technical proficiency, improving their operating performance, and decreasing the operation time.

The use of virtual reality (VR) and immersive VR (iVR) in surgical training has become increasingly popular due to the advancements in technology (4, 5).

iVR provides a more realistic and immersive experience compared to conventional VR, allowing trainees to simulate surgical procedures in a more realistic operating room environment.

This technology has been shown to improve technical proficiency, decrease operation time, and increase the attractiveness and degree of participation in surgical training (6–8).

Consequently, this system can provide an immersive surgical experience that enhances the knowledge and skills required for a given surgical procedure.

iVR has the advantages of conventional VR and operates on low-cost, portable, and commercially available hardware. Unlike conventional VR, immersion in iVR is provided as an uninterrupted, scaled environment capable of simulating the full magnitude of sensory stimuli present in the OR (5).

With the COVID-19 pandemic and physical distancing, VR may play a crucial role in providing surgical education to medical students whose regular clerkship experiences have been suspended (9).

Previous studies have demonstrated the use of VR as an advantageous tool in anatomy education and residency training for medical students (10–13).

The development of iVR training systems for orthognathic surgery is particularly important given the challenges of this type of surgery, including complex anatomical structures, limited surgical field of intraoral approach, and the use of various surgical instruments.

While previous studies have focused on specific procedures, such as the application of a reciprocating saw in Le Fort I osteotomy, the use of iVR for double jaw orthognathic surgery has not been explored extensively.

Therefore, we developed an iVR training system for double jaw orthognathic surgery. We assessed the validity of this system and explored the possibility of using it as an additional training tool for orthognathic surgery.

## Material and methods

Seven senior surgeons (experts) and seven fifth-year medical students (novices) were enrolled from our hospital after obtaining institutional review board approval and written informed consent for participation.

Each participant completed a pretest questionnaire reporting their age, sex, and prior exposure (cases observed) and experience (cases performed) with orthognathic surgery as well as any previous experience with VR devices.

All participants tested the iVR system for approximately 1 h, completing the training mode and assessment mode of bimaxillary orthognathic surgical procedure.

**Figure 1:**
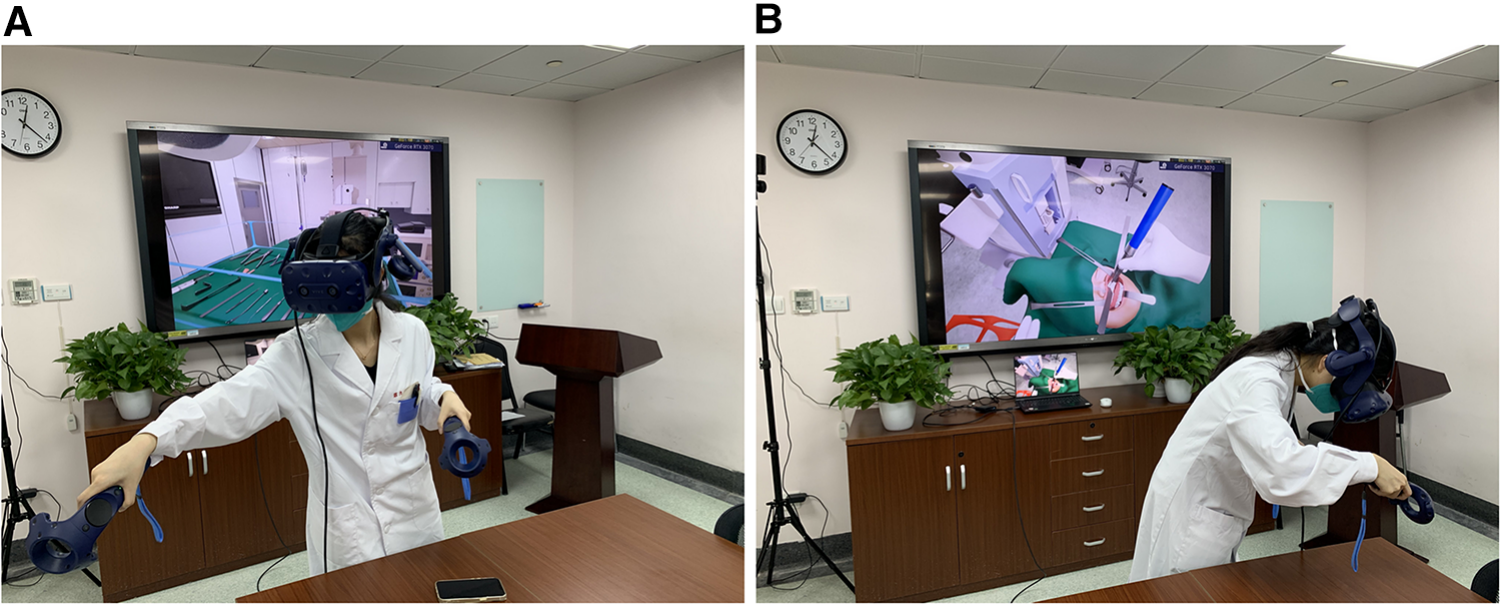
(**A**) the trainee is picking instrument from the table. (**B**) The trainee is doing the osteotomy with reciprocating saw.

**Figure 2:**
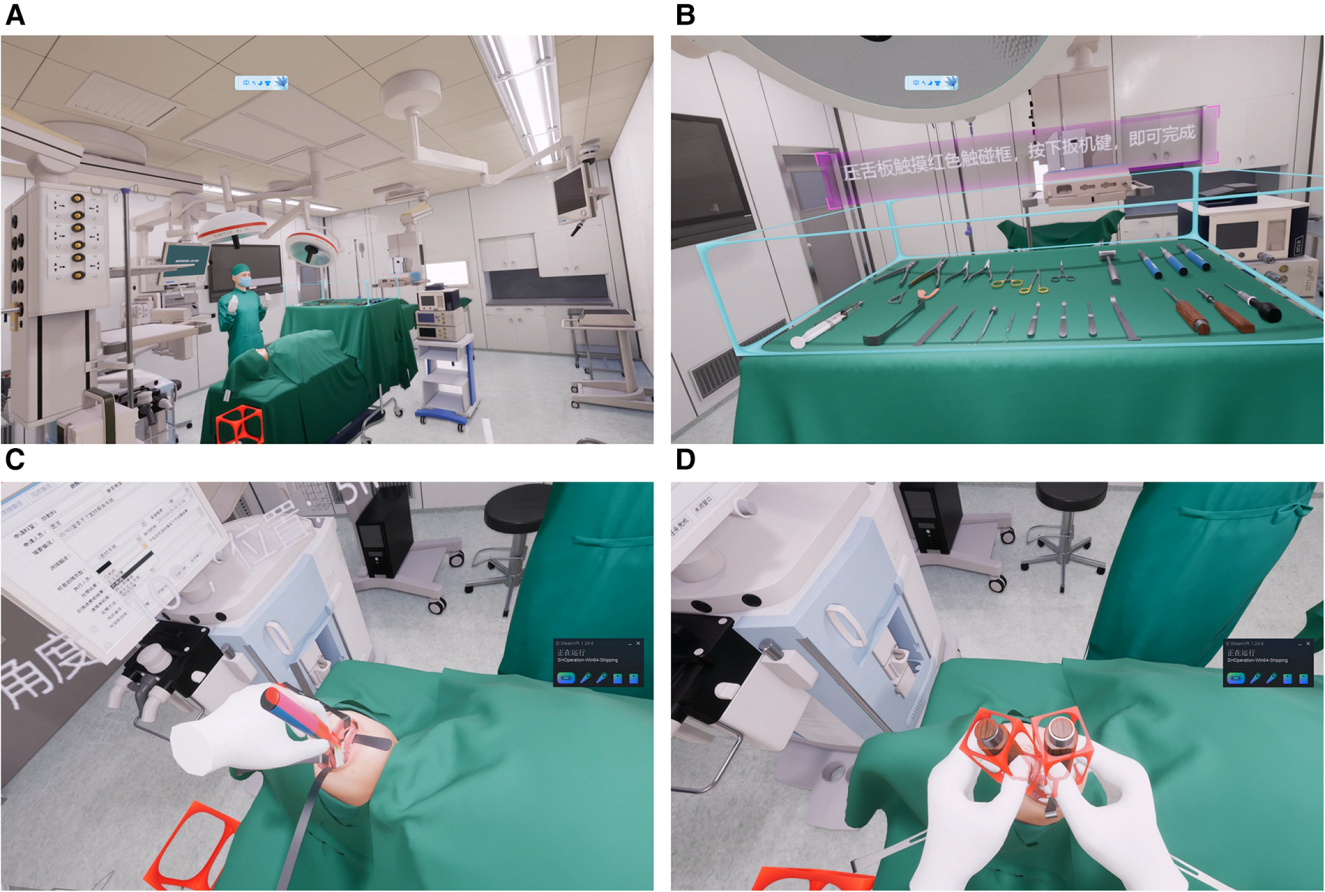
(**A**) the simulated operating room. (**B**) Instrument table. (**C**) Le Fort I osteotomy. (**D**) BSSRO.

During their first VR session, each participant completed a tutorial on general orientation in the VR space (Figures 1, 2).

A survey of user experience was administered at the end of the course (Table 1).

**Table 1:** The content of the questionaire.

The surgery is shown accurately in the system.
All the steps of the surgery are covered in the system.
The anatomy of the skull in the system is accurate.
The anatomy of the soft tissue in the system is accurate.
The instruments in the system are shown accurately.
The environment of the OR is shown accurately.
The scoring system is set properly.
I feel dizzy during the test.
I found this system interesting.
This is very helpful to my learning LFI surgery.
This is very helpful to my learning BSSRO surgery.
This application can increase my interest in learning.
This application can be used as a supplementary to traditional surgical training.
I hope more procedures can be made into VR.

The survey included 14 questions asking participants to rank their level of agreement regarding the use of VR on a 5-point Likert scale (1 = strongly disagree, 2 = disagree, 3 = neutral, 4 = agree, and 5 = strongly agree).

The questionnaire consisted of two sections. The first section focused on the experience and application of the VR system. The second section focused on the surgical content, including anatomical accuracy, integrity of the surgical procedure, and fidelity of the OR and surgical instruments.

### Development of the VR system

We developed an iVR orthognathic surgical training system that places operators in a virtual OR and allows them to use surgical instruments on a virtual patient to perform bimaxillary orthognathic surgery.

The HMD device used in this study was HTC VIVE Pro 2 (HTC Corporation, Taiwan). The virtual operation room was created based on a real OR captured using a 360° camera.

Virtual models of the surgical instruments required in our clinical practice were generated using obverse design. The patient model was generated based on the computed tomography (CT) data and optical scan of dental casts of a real patient with skeletal class III malocclusion.

Subsequently, the entire surgical procedure was scripted using Unreal Engine 4 (Epic Games, Inc. NC, USA) to generate the operation flow of the VR training system.

The maxillary surgical procedures included incision, Le Fort I osteotomy, intermaxillary fixation with the intermediate splint, and maxilla fixation. The mandibular surgical procedures included incision, bilateral SSRO, intermaxillary fixation with the final splint, and mandible fixation.

We developed two modes in this system: training and assessment. The training mode involved VR guidance to demonstrate the next surgical step or instrument and the correct instrument positions to the trainee. Voice prompts of the surgical procedures were integrated in the system.

In each step, a virtual assistant would hand out the required surgical instrument to the trainee, and the placement of each instrument was depicted as a phantom for further guidance.

In the assessment mode, the surgical instruments were placed on the instrument table. The trainee was required to select the appropriate instruments required and place them correctly to initiate the next surgical step without any guidance.

The following four measures were used to evaluate the operator performance in the iVR training system: procedural duration (minutes), number of instrument selection errors, instrument position and angular errors, and number of prompts required to progress to the next step (triggered by the operator). The assessment record was available to the trainee at the end of the virtual procedure.

## Results

All 14 participants completed the test and questionnaire. The mean age of the experts and novices was 38.6 years and 22.1 years, respectively. The expert group comprised six men and one woman, whereas the novice group comprised two men and five women. The experts had a mean orthognathic surgical experience of 7.3 years, with 371 cases observed and 187 cases performed, whereas the novices had no previous orthognathic experience, with an average of 4.4 cases observed. The medical students observed at least one bimaxillary orthognathic surgery before completing the questionnaire. One expert and one novice had prior experience with VR devices.

**Figure 3:**
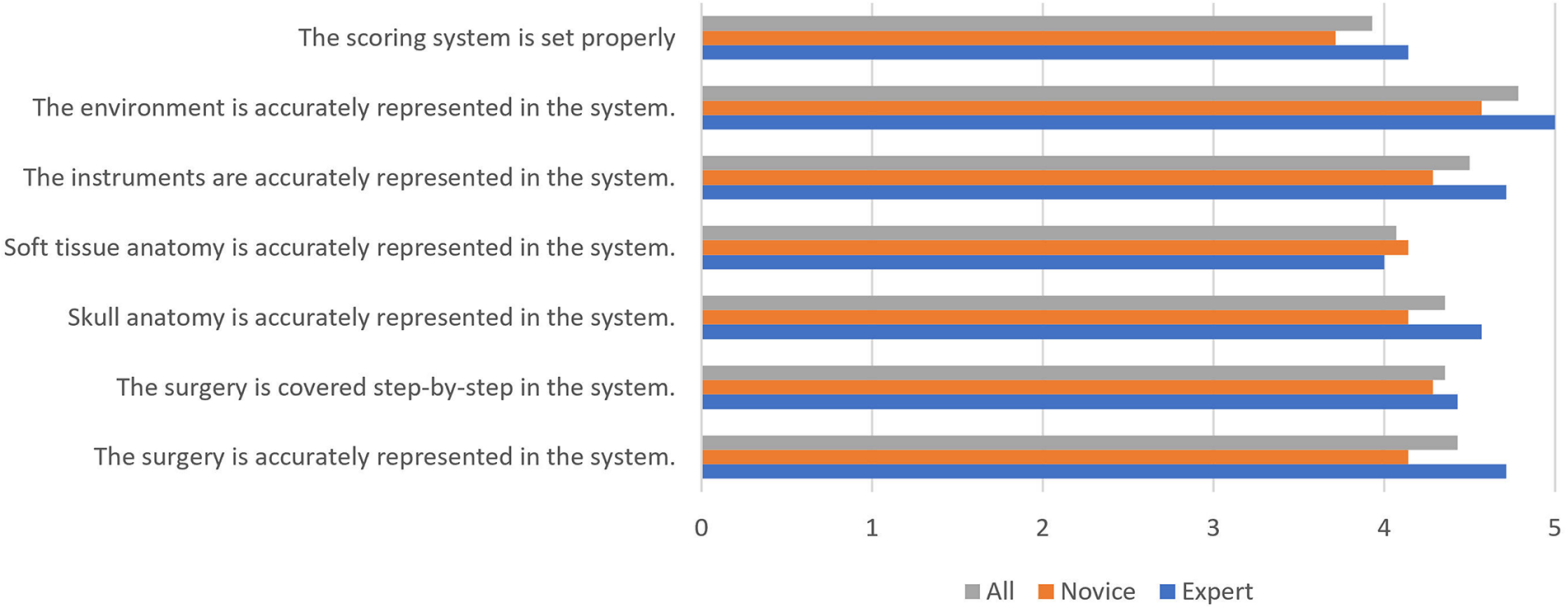
The first section of the questionnaire.

The mean score of the first section was 4.35 out of 5, demonstrating strong agreement to the fidelity of the system. The answers to each question are shown in Figure 3.

**Figure 4:**
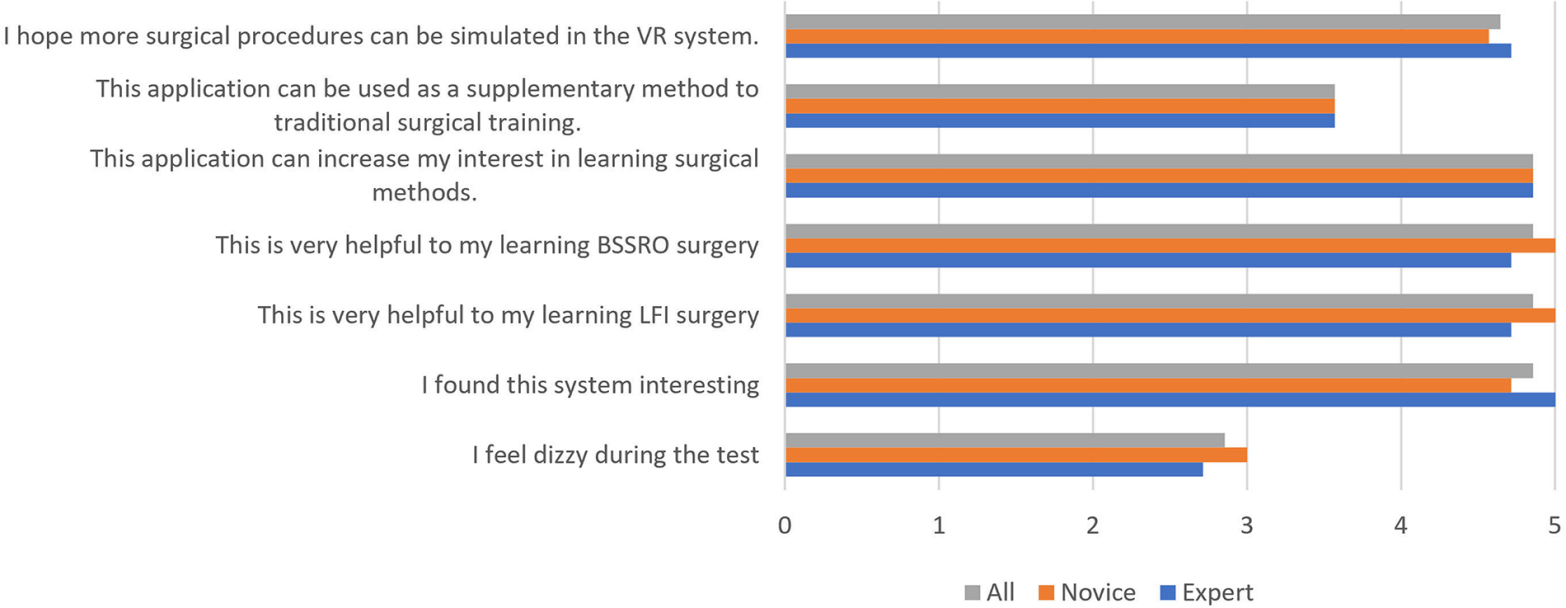
The second section of the questionnaire.

The mean score of the second section was 4.40 out of 5. The answers to each question are shown in Figure 4. Most participants experienced some degree of dizziness or fatigue after the 1-h test. All novice participants strongly agreed that this application was helpful to their learning of the surgical technique (5 out of 5).

## Discussion

In general, learners initially go through a cognitive stage to become acquainted with the task and learn the prerequisite steps. Subsequently, in the integrative stage, the specific tasks become familiar. Finally, in the autonomous stage, the overall task performance becomes smooth and efficient (14).

Junior surgical residents often prepare themselves for surgical procedures by studying technical guides and watching videos of the procedure (1). The results of this study demonstrated that iVR was realistic and useful in orthognathic surgical training.

Most participants agreed that our iVR training system was a useful learning tool, which could enhance their understanding of surgical procedures and interest in orthognathic surgery.

Dizziness was the main complaint regarding the iVR system. In this study, participants experienced no (36%), slight (28%), and moderate (36%) motion sickness.

The goal of VR is to replace the physical world with a virtual world and render the 3D environment immersive, semi-immersive, or no immersive.

In an immersion system, users find themselves in a complete virtual environment through its sensory output devices, which may be visual (HMD), audio, or haptic. HMD is more widely used because this application is more flexible and requires a smaller space than audio or haptic devices.

The essential points of VR surgery are the 360° experience, close-up stereoscopic visualization, and 3D interaction (15, 16).

VR may offer a useful adjunct to conventional training methods, which may help residents learn the procedural workflow and specific application of surgical instruments to perform surgical procedures.

The application of VR technology in clinical surgery enables users to efficiently learn surgical procedures that can be subsequently applied safely at any point throughout the training process, and it is especially useful before performing an operation for the first time. The implementation of VR for clerkship preparation or as a supplement to surgical rotation can improve students' knowledge and clinical skills (17).

As a common maxillofacial surgical procedure, orthognathic surgery involves a complex interplay of cognitive (maxillofacial anatomy and surgical instrument application), combined (instrument placement and surgical process), and technical (saw control and soft tissue handling) skills.

However, the intraoral approach greatly reduces intraoperative observation due to the limited surgical field. Thus, orthognathic surgery is an appropriate example of a complex and highly skillful surgical procedure, where there is an opportunity to demonstrate the effectiveness of assistive technology on surgical performance.

Several previous studies have explored the feasibility of using VR techniques to train inexperienced oral maxillofacial surgeons. For instance, Pulijala et al. developed a VR training tool for orthognathic surgery based on 360° and stereoscopic videos of orthognathic surgery (18, 19). However, the surgical procedures were mainly presented using stereoscopic videos instead of virtual model interaction.

Wu et al. presented a VR-based training system primarily used for application in Le Fort I osteotomy by reconstructing virtual models from CT data and using virtual instruments built from laser scanning data (20). The system mainly focused on the haptic force feedback simulation rather than on complete surgical steps and procedures.

Arikatla et al. presented methods to simulate bone drilling and cutting with low computational cost and high-fidelity haptic feedback in BSSO (21).

Furthermore, virtual rigid skull and jaw models without soft tissues have been commonly used in OMFS simulators (22). However, most of the abovementioned studies focused on the haptic feedback algorithm of bone drilling and cutting. Therefore, the VR training system used in those studies could not provide an immersive simulation of the entire surgical procedure and operation environment.

VR applications, such as Touch Surgery (Kinosis, London, United Kingdom) and CIVA (BioDigital, New York, USA), have been used to teach surgical procedures using the cognitive task analysis theory. The entire surgical procedure could be divided into a series of cognitive steps, such as decision making, problem solving, memory, attention, and judgment. Consequently, trainees can increase their familiarity with surgical tools and anatomical landmarks as well as become accustomed to the most common errors committed during any given surgical procedure (23, 24). However, the immersive experience and interaction with virtual objects in these web-based applications remains limited.

Therefore, we developed an iVR surgical learning system for orthognathic surgery, aiming to integrate the entire procedure of orthognathic surgery into the system with high fidelity. The operation room in our iVR system was created with rich details based on a real OR to provide the operator with an immersive learning environment.

Virtual models of all surgical instruments required during real-world operation were generated and could be freely selected and applied. The correct position and angle of several specific instruments were marked and recorded into the system.

There is a distinct gap between isolated skill training and performing the entire surgical procedure in the OR. Our procedural VR system helps the trainee combine each of these little steps simultaneously and complete the complex orthognathic procedure efficiently.

However, there are also limitations and challenges that need to be addressed. The iVR system's simulation was primarily focused on visual details, haptic feedback and physical interaction experiences are still lacking. VR dizziness and resolution ratio of HMD were the most common complaints by the participants, which could be improved in the future with continuous development of the iVR technique and commercial VR devices.

Additionally, there were also limitations of this study. The study had a limited sample size, and it lacked a control group for comparison.

Additional research is necessary, utilizing randomized controlled trials with a more substantial sample size. Future studies should evaluate the utility of VR training at various levels of surgical education and residency training.

iVR technology has the potential to revolutionize surgical training and education by providing standardized and cost-effective training, objective assessment, and multi-user capabilities. As the technology continues to develop and improve, it may become an increasingly valuable tool in surgical training and education.

## Data Availability

The original contributions presented in the study are included in the article/Supplementary Material, further inquiries can be directed to the corresponding author/s.
